# Changes in sulfur in soybean rhizosphere soil and the response of microbial flora in a continuous cropping system mediated by *Funneliformis mosseae*

**DOI:** 10.3389/fmicb.2023.1235736

**Published:** 2023-08-23

**Authors:** Yizhi Mao, Donghao Chang, Xiaoying Cui, Yunshu Wu, Baiyan Cai

**Affiliations:** ^1^Engineering Research Center of Agricultural Microbiology Technology, Ministry of Education and Heilongjiang Provincial Key Laboratory of Ecological Restoration and Resource Utilization for Cold Region and Key Laboratory of Molecular Biology, College of Heilongjiang Province and School of Life Sciences, Heilongjiang University, Harbin, China; ^2^Hebei Key Laboratory of Agroecological Safety, Hebei University of Environmental Engineering, Qinhuangdao, China

**Keywords:** soybean, continuous cropping soil, *Funneliformis Mosseae*, sulfur, response of flora, sulfur functional gene

## Abstract

Soybean is an S-loving crop, and continuous cropping might cause soil sulfur shortage. The primary objectives of this study are to determine whether *Funneliformis* mosseae (*F. mosseae*) can enhance the content of available S in S-deficient soil and thereby improve the sulfur utilization rate in soybean. The experiment used Heinong 48 (HN48), a soybean variety with a vast planting area in Heilongjiang Province, and *F. mosseae* was inoculated in the soil of soybean that had been continuously cropped for 0 and 3 years. The results of the barium sulfur turbidimetric assay show that the sulfur content in the soil and soybean was reduced by continuous cropping and increased by inoculation with *F. mosseae*; the results of the macro-genome sequencing technology, show that the diversity and abundance of bacteria in the soil was decreased by continuous cropping and increased by inoculation with *F. mosseae*. The sulfur-oxidizing bacteria (SOB) activity and sulfur-related gene expression levels were lower in the continuous crop group compared to the control group and higher in the *F.mosseae*-inoculated group compared to the control group. Continuous cropping reduced the sulfur content and ratio of soybean rhizosphere soil, affecting soil flora activity and thus soybean growth; *F. mosseae* inoculation increased the sulfur content of soybean root-perimeter soil and plants, increased the diversity and abundance of rhizosphere soil microorganisms, increased the expression of genes for sulfur transport systems, sulfur metabolism, and other metabolic functions related to elemental sulfur, and increased the species abundance and metabolic vigor of most SOB. In summary, continuous cropping inhibits soil sulfur uptake and utilization in soybean while the inoculation with *F. mosseae* can significantly improve this situation. This study offers a theoretical research foundation for using AMF as a bio-fungal agent to enhance soil sulfur use. It also supports the decrease of chemical fertilizers, their substitution, and the protection of native soil.

## 1. Introduction

Soybean is a crop that enjoys sulfur. Sulfur plays critical roles in soybean growth, development and metabolism. Sulfur not only promotes protein synthesis and accumulation of protein in soybean grains, but it also promotes fat formation (Cao, 2016). Sulfur deficit in farmland is widespread due to the ongoing improvements in the multiple cropping index and decrease in the quantity of sulfur returned to farmland soil, resulting in a steady reduction in available soil sulfur for crops. Sulfur insufficiency has emerged as a major issue in agricultural production (Guo et al., 2021). Numerous studies have shown that Arbuscular mycorrhizal fungi (AMF) improve crop nutrients and water absorption through their extensive exophytic hyphal system (Qi et al., 2021), improve soil physical and chemical properties in the plant rhizosphere, and increase soil fertility (Fernandes et al., 2021). To address the soil sulfur deficiency in continuously cropped soybean, the characteristics of AMF that can promote the absorption of soil mineral elements and repair soil with continuous cropping obstacles were examined in this study to determine how to improve the utilization rate of soil sulfur by AMF (Deng et al., 2021; Guo et al., 2021). This study has significant theoretical and practical implications for modifying the soybean production mode, ensuring high-quality green organic soybean production, and increasing the yield quality and farmland ecosystem. Previous studies on the relationship between AMF and S found that after soil inoculation with SOB, the utilization of sulfur increased significantly (Joshi et al., 2021; Malviya et al., 2022). When AMF was co-inoculated with SOB, it could significantly increase soil fertility, improve soil physicochemical properties, increase soil enzyme activity, and promote plant growth (Heydari and Pirzad, 2021), indicating that AMF and SOB have good synergistic ability. Sulfur can be transmitted from AMF to plants, and sulfur and sulfur-containing organic compounds can be carried via the mycorrhizal pathway (Deng et al., 2021). Under low-sulfur situations, AMF improve available sulfur absorption and assimilation and so increase plant sulfur content (Crespo et al., 2021). We used *F. mosseae* microbial inoculum to continuously cropped soybean soil to address the shortage of sulfur, which impacts soybean growth, yield, and quality. How *F. mosseae* boosted soil sulfur absorption by continuously cropped soybean was investigated in light of the breakthrough effect of *F. mosseae* on the supply of sulfur in continuously cropped soybean soil. Following the inoculation of *F. mosseae* microbial inoculum, the soil’s total sulfur, available sulfur, and soybean biomass were analyzed in this study on the basis of soil that had been continuously cropped with soybean for 3 years. The reaction of soil SOB was discovered by the metagenomic analysis of soil samples from the soybean rhizosphere.

## 2. Materials and methods

### 2.1. Fundamental state of the sample plot and test soil

From May to September 2021, the experiment was carried out at the Sugar Research Institute of Harbin Institute of Technology (46°40′N, 130°10′E). Farmland soil sample plots that had been continuously cropped for 0 years (corn in the previous year, soybean in the first year) and 3 years (corn in the third consecutive year) were selected. Topsoil was obtained at random form the target plot as potting soil for the potting experiment. Table 1 shows the initial physical and chemical indices of the experimental soil, which is chernozem in cold cropland at 45 degrees north latitude.

**Table 1:** Basic physical and chemical indices of soil in the sample plots.

Physicochemical indexes	Electrical conductivity (μS·cm^−1^)	Total N (g·kg^−1^)	Total K (g·kg^−1^)	Total P (g·kg^−1^)	Total S (mg·kg^−1^)
0 year soil	513.45	1.63	25.4	5.2	435.68
3 years soil	833.96	1.54	24.8	4.8	422.19
Physicochemical indexes	pH	Available N (mg·kg^−1^)	Available K (mg·kg^−1^)	Available P (mg·kg^−1^)	Available S (mg·kg^−1^)
0 year soil	7.5	129.1	207.2	13.27	14.85
3 years soil	7.4	127.9	204.3	12.88	12.78

### 2.2. Cultivars and fungal strain

#### 2.2.1. Soybean variety

HN 48 (protein content: 44.71%; fat content: 19.05%) is a high-protein soybean cultivar that is frequently cultivated in Heilongjiang Province.

#### 2.2.2. Strain to fungal inoculum

*Funneliformis mosseae* microbial inoculum (25 spores/g) was purchased from the Institute of Plant Nutrition and Resources, Beijing Academy of Agricultural and Forestry Sciences, and *F. mosseae* mixed microbial inoculum was obtained through *Medicago sativa* propagation as the host plant.

### 2.3. Experimental design

To more precisely quantify the components in rhizosphere soil, this experiment adopted a pot culture design, placing soybean soil that had been continuously cropped for 0 and 3 years into pots with an upper diameter of 25 cm and a height of 20 cm.

The potted plants were randomly divided into four groups and treated as follows: continuous 0-year soil (C); continuous 0-year soil and inoculated with *F. mosseae* fungicide (T); continuous 3-year soil (CC); and continuous 3-year soil and inoculated with *F. mosseae* fungicide (CT).

5 kg of soil was sown in each pot in late May 2021, and 45 g of *F. mosseae* microbial inoculum was infected; three soybean plants were retained, 20 pots were used in each treatment, and 80 pots were used in total. Samples were collected 30, 45, 60, 75, and 90 days after soybean emergence. When the treatment group’s infection rate peaked, three duplicate samples from four treatments were exposed to metagenome sequencing.

### 2.4. Experimental method

#### 2.4.1. Collection and processing of samples

##### 2.4.1.1. Soil physical and chemical indexes determination sample collection

Soybean rhizosphere soil was collected every 15 days after the emergence of soybean seedlings, each treatment randomly selected three pots of potted soybeans, each pot of soybeans to collect soil samples in the range of 1–5 cm of the roots, each pot to collect 200 g of rhizosphere soil and mix thoroughly, each treatment group to collect three parallel samples, a total of 12 soil samples were collected in the four treatment groups. After the soil samples were dried naturally, they were ground and crushed, and screened with a 60-mesh sieve to obtain 150–170 g of sieved soil. 5 g of sieved soil was used to measure the soil pH value, 10 g of sieved soil was used to measure the soil conductivity, and 100 g of sieved soil was used to measure the soil sulfur index, and the rest of the soil was placed in the refrigerator at −20°C to keep a backup.

##### 2.4.1.2. Measurement of soybean growth

Each time we collected samples for the measurement of soil physical and chemical indexes, the plants were carefully separated from the soil, washed and dried, and then the basic growth indexes of soybean plants, such as plant height, root length, and dry weight, were measured separately.

##### 2.4.1.3. Sample collection for the determination of AMF infestation rate in soybean root system

For each sample collection for the determination of soil physical and chemical indexes, 100 sections of 1 cm fibrous roots were cut from the root system of each pot of soybeans, and stored in a water bath at room temperature for the determination of the infestation rate of AMF.

##### 2.4.1.4. Sample collection for the determination of sulfur content of soybean functional leaves

Each time when collecting samples for the determination of soil physical and chemical indicators, each pot of soybean functional leaves were collected, washed and placed in a blower drying oven at 80°C heated for 30 min, and then cooled down to 60°C to dry the water, crushed for the measurement of the S content of the functional leaves of soybeans.

##### 2.4.1.5. Macrogenome sequencing sample collection

Soybean rhizosphere soil was collected when the AMF infestation rate in the soybean root system reached its peak (75 day after soybean seedling emergence). During sample collection, three pots of experimental soybeans were randomly selected for each treatment, and soil samples were collected from the 1–5 cm range of the roots in each pot. 20 g of rhizosphere soil was collected from each pot and mixed thoroughly, and three parallel samples were collected from each treatment group; a total of 12 fresh soil samples were collected from the four treatment groups. From each fresh soil sample, 10 g of fresh soil was weighed and treated with liquid nitrogen, and then placed in a foam box with dry ice to protect it from light, and then sent to a biotechnology company for macro-genome sequencing analysis.

#### 2.4.2. *Funneliformis osseae* infection rate determination

Phillip and Hayman’s acid magenta staining method was used to determine the infection rate of AMF in the root system (Kormanik et al., 1980). Based on microscopic analysis, samples were gathered and tallied, and the percentage of infested roots was estimated. BX41 optical microscope (OLYMPAS) was used for microscopic investigation.

#### 2.4.3. Measurement of soil pH value

The pH value of the soil was determined using the electrode method and a standard pH meter, the FE20 pH Meter (METTLER TOLEDO). The standard solution was calibrated three times before to the experiment.

#### 2.4.4. Determination of available sulfur, total sulfur in soil, and sulfur in leaves of soybean

Barium available sulfur turbidimetry was used to determine the total sulfur and available sulfur levels of the test sample (Su and Gelius, 2020; Yue et al., 2021; Zhang et al., 2021; Zhao et al., 2021, 2022). According to the standard curve and the spectrophotometric value, the content of sulfur in the sample is calculated, the spectrophotometer adopts UV-min1240 (Shimadzu).

##### 2.4.4.1. Standard curve

Pipette 50 mg/L S standard solution, 0.00, 2.00, 4.00, 6.00, 8.00, and 10.00 mL into a 50 mL volumetric flask, add glacial acetic acid 5 mL, H_3_PO_3_ 1 mL, 0.5% gum Arabic 1 mL, and make up to 50 mL; after shaking well, transfer to a 100 mL beaker, add BaCl_2_ crystals 1 g, stir on a magnetic stirrer for 1 min, within 30 min, in a 3 cm colorimetric bath, read the absorbance at 440 nm with a spectrophotometer; finally, take mg/L S as the horizontal coordinate, the corresponding absorbance as the vertical coordinate, and plot the standard curve on the coordinate paper.

##### 2.4.4.2. Sample determination

1.0000 g of air-dried soil sample was weighed to 0.5 mm in a 500 mL beaker, 2 mL of Mg (NO_3_)_2_ solution was added and evaporated to dryness at 70°C on an electric hotplate. The residue was placed in a high temperature electric oven at 300°C overnight. After cooling, 5 mL of HNO_3_ was added, the surface dish was covered and decocted on a water bath for 2.5 h, taking care not to lose HNO_3_. After cooling, dilute with water to about 20 mL, filter into a 50 mL volumetric flask with filter paper, wash several times, and dilute to about 40 mL. Add glacial acetic acid 5 mL, H_3_PO_3_ 1 mL, 0.5% gum Arabic 1 mL, make up to 50 mL. Transfer to a 100 mL beaker, add BaCl_2_-2 H_2_O grains 1.0 g, stir on a magnetic stirrer for 1 min, within 30 min load into a 3 cm colorimetric bath, turbidimetric at 440 nm using a spectrophotometer, and do the reagent while determining the sample blank test.

#### 2.4.5. Metagenome sequencing

For metagenome sequencing, DNA was collected from soil samples (Aymé et al., 2021; Ramírez-Fernández et al., 2021; Chamberlain et al., 2021).

The sample’s metagenome sequencing involved the following steps: the metagenome sequencing of the samples was finished; Sequencing data processing and statistics, including the low-quality data filtering, data output, and quality control statistics, were carried out; The metagenome was assembled using MEGAHIT software, contig sequences shorter than 100 bp were screened, and the assembly results were evaluated using QUAST software. To identify the coding region in the genome and perform gene prediction, Metagene Mark software (http://exon.gatech.edu/meta_gmhmmp.cgi, Version 3.26) was used with default parameters; Cd-hit software (version 4.6.6 of http://www.bioinformatics.org/cd-hit/) was chosen to remove redundancy; the similarity threshold was set to 95%, and the coverage threshold was set to 90%.

#### 2.4.6. KEGG annotation

By BLAST matching the protein sequences of nonredundant genes with those included in the KEGG database, the most similar sequence in the KEGG database was obtained, and the annotation information of this sequence was the annotation information of the corresponding gene in the sequenced genome (Kölbl et al., 2019; Liu et al., 2020; Li J. et al., 2021; Coutinho et al., 2021; Hu et al., 2021; Rai et al., 2021; Ma et al., 2022).

#### 2.4.7. Statistical analysis

By analyzing the functional genetic makeup of different samples, functional principal component analysis (PCA) indicated differences and distances between samples (Cao et al., 2016; Blaise et al., 2021; Calegario et al., 2021; Makowska et al., 2021). Permutational multivariate ANOVA (PERMANOVA, also known as Adonis analysis) was used to examine the degree of explanation of differences among samples by different grouping factors and their statistical significance using a permutation test (Deng et al., 2021; Li B. B. et al., 2021; Ramírez-Fernández et al., 2021; Yang et al., 2021). Using R’s vegan package and box-line plots based on grouped distance matrices, the two distance matrices produced from βdiversity analysis were subjected to PERMANOVA (Xie et al., 2021). Parametric statistics were chosen to make inferences or perform hypothesis tests on the overall parameters using sample indicators. Parametric tests for differences between the two groups were conducted using ANOVA (Farhangi-Abriz et al., 2021; Guo et al., 2021; Jalal et al., 2021), NMDS analysis, SOB differential species analysis, and RDA etc. (Kelly et al., 2021; Miao et al., 2021; Schwalbert et al., 2021).

One-way ANOVA analyzed data, NMDS using IBM SPSS Statistics 25 software to determine the significance of the differences between the different treatment groups (*p* < 0.05); Python software was used to analyze the data through α-diversity (the Chao1 index and the ACE index) and β-diversity (the Bray–Curtis-Curtis algorithm and the binary Jaccard algorithm), and significant differences were found among different processing groups (*p* < 0.05); Origin 2022, Python-3.6.0 and R-4.1.3 were used for correlation and significance analysis (*p* < 0.05), and data visualization analysis was performed for the measured indicators.

## 3. Results

### 3.1. AMF infestation rate and soil pH

**Figure 1:**
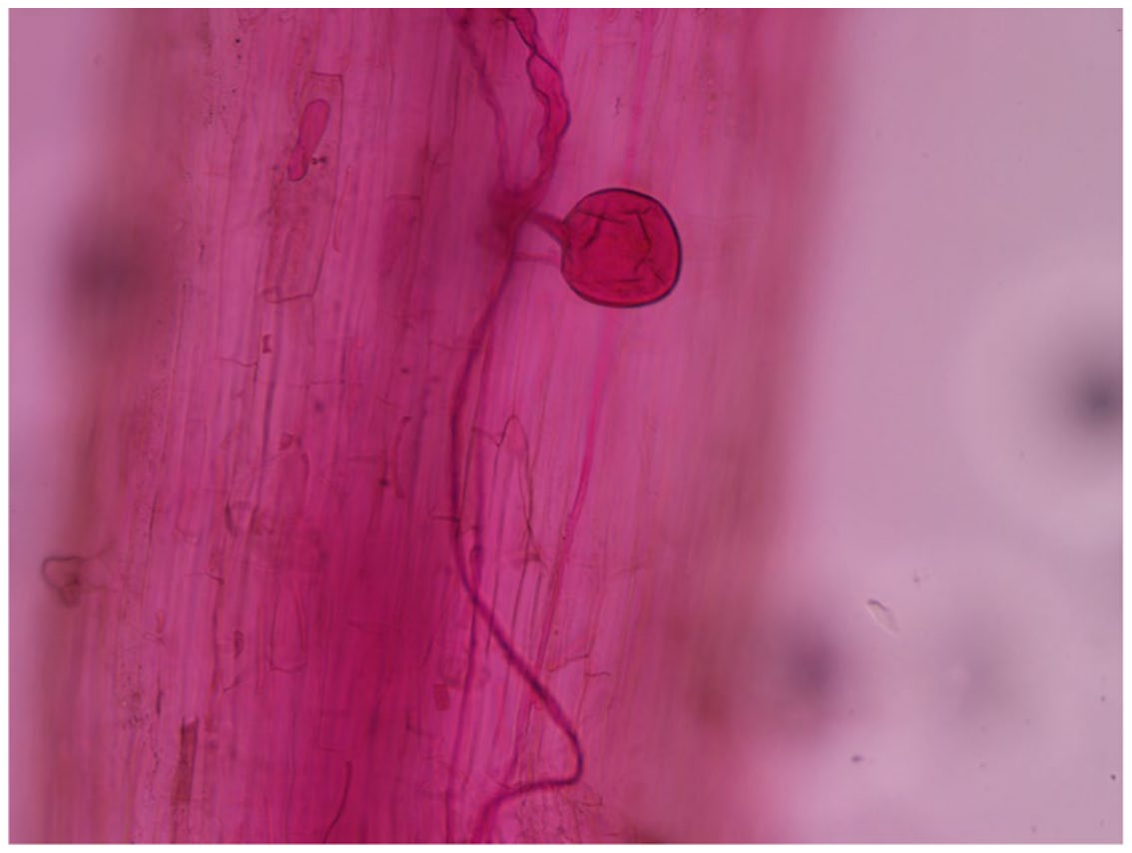
Colonization diagram of *Funneliformis mosseae* in the roots of soybean.

**Figure 2:**
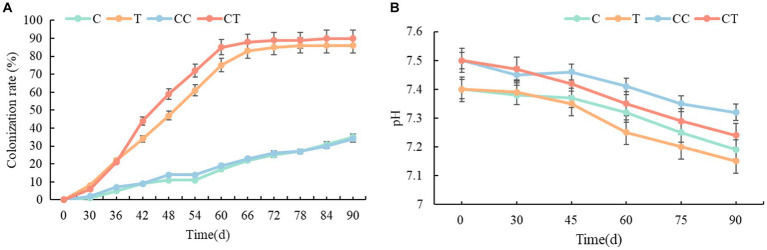
**(A)** Test of the infection rate of soybean roots with *Funneliformis mosseae*; **(B)** Test of the soil pH value.

Arbuscular mycorrhizal fungi infection was discovered in the roots of the four treatments 30 days after soybean emergence, and evident AMF vesicle structures appeared 45 days after emergence (Figure 1). Figure 2A depicts the changing trend of the AMF infection rate of the four treatments during the soybean growing period. The root infection rate of the T and TC groups peaked out approximately 70 days and gradually stabilized, whereas the root infection rate of the C and CC groups continuously rose as the growth period lengthened. Affected by the wild AMF in the soil, AMF infection was also detected in the roots of groups C and CC, with the highest infection rate of around 35%, which did not substantially increase with the extension of the soybean growth period (*p* < 0.05). The highest infection rate of groups T and CT was nearly 90%, which roughly corresponded to the growth curve of the soybean plants. The infection rate of AMF in soybean roots was usually considerably greater than that of the uninfected treatment (*p* < 0.05). The findings revealed that as soybean grew and developed, the infection rate of AMF in soybean roots would gradually increase, and wild arbuscular mycorrhizal fungi also existed in the natural environment.

The soil pH of the four treatments steadily lowered as the soybean grew and developed (Figure 2B), which was thought to be due to an increase in soluble acidic compounds in the soil caused by the physiological activities of soil rhizobia and SOB. Overall, the pH in the continuous cropping group was lower than in the noncontinuous cropping group during the growing period, and it is anticipated that soil acidification is becoming a concern as a result of continuous cropping operations. The pH of the inoculated group gradually fell when compared to the uninfected group. It is hypothesized that AMF can improve rhizosphere soil by increasing circulation and aeration efficiency of soil mineral components and alleviating the acidic reaction process under hypoxia. The results show that continuous cropping of soil will lead to soil acidification, and that AMF mycorrhizal results can effectively inhibit the acidification process of rhizosphere soil.

### 3.2. Analysis of sulfur content and speciation transformation

#### 3.2.1. Sulfur content and form transformation in soybean rhizosphere soil

**Figure 3:**
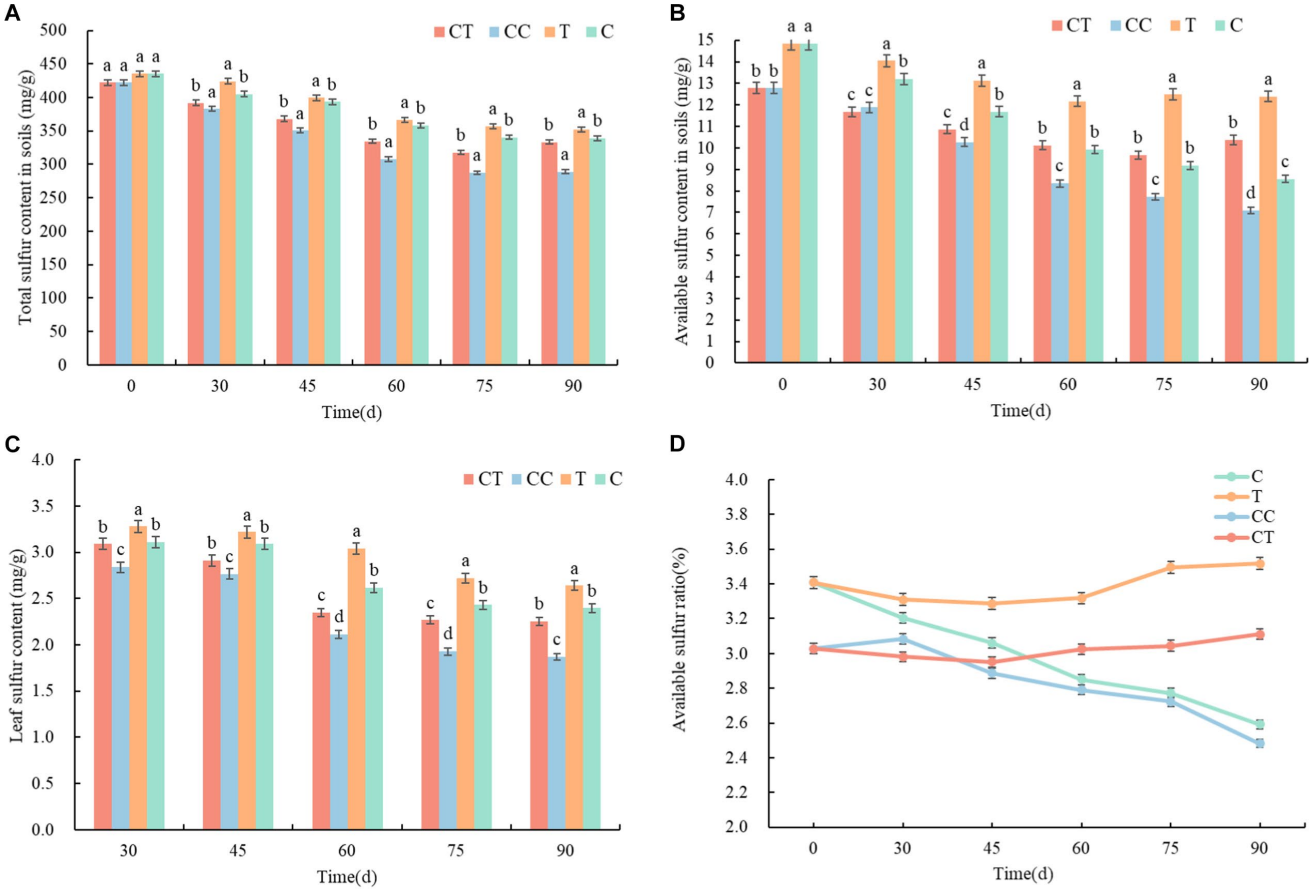
Changes in sulfur content and morphology between treatments. **(A)** Total sulfur in the rhizosphere soil; **(B)** Available sulfur in rhizosphere soil; **(C)** Sulfur content in soybean leaves; and **(D)** Proportion of available sulfur in rhizosphere soil. These lowercase letters (a, b, c, d) indicate the meaning of significant differences between the treatment groups.

The content of available sulfur in soybean rhizosphere soil has a direct impact on the absorption of S by the root system and the growth process of soybean plants. The total sulfur content in the soil around the roots of continuously cropped soybean was detected and analyzed, and the total sulfur content in the four treatments steadily reduced (Figure 3A) over the growth period of the soybean. The total S content of the C and T treatment groups’ noncontinuous cropped soil was significantly higher than that of the CC and CT treatment groups’ continuously cropped soil, and the total sulfur content in the soil near the roots of the CC group was the lowest. The results suggest that continuous cropping reduces soil sulfur content, and a low sulfur environment reduces the richness and abundance of soil SOB (*p* < 0.05).

According to an analysis of the soybean growth process, the difference between CC and CT was minimal at 30 days after emergence, but there was a significant difference between the T and C groups. It was speculated that *F. mosseae* was in its own growth and development stage at the initial stage of inoculation and that the soybean root infection rate was low, which slightly impacted soybean growth and metabolism, and it was difficult to demonstrate its superiority when the growth of soybean roots was limited. The available sulfur in group T was higher than in group C 60 days after emergence. There was a significant difference between the available sulfur in CC and CT. According to the trend of the AMF infection rate of the soybean roots, *F. mosseae* had a limited role in noncontinuous cropped soil, but it significantly improved the supply capacity of sulfur surrounding the roots in continuously cropped soil and lowered the total sulfur level in the soil (*p* < 0.05). The soybean grain growth period occurs 75–90 days after the emergence of soybean.

During this time, photosynthetic products are delivered to soybean grains, and root development and sulfur absorption stabilize. The CT group’s total sulfur content in the soil was considerably higher than the CC group’s (*p* < 0.05). It is hypothesized that *F. mosseae* inoculation improved soil flora structure, increased soil SOB abundance, improved metabolic function, promoted sulfur enrichment and inclusion by SOB groups, and lowered the quantity of sulfur in irrigation water. The total sulfur level in the uninoculated soil exhibited a substantial decrease trend. The total sulfur content of inoculated soil fell marginally compared to nonincubated soil and rose at the peak of the infection rate. The overall sulfur concentration was nearly same in groups C and CT. The results showed that *F. mosseae* increased plant sulfur uptake, decreased sulfur concentration near roots, and aided in the conversion of total sulfur to effective S.

The available sulfur content in the soybean rhizosphere soil of the noncontinuous cropping (C and T) groups was slightly higher than that of the continuous cropping (CC and CT) groups at 30–60 days after emergence, and it was significantly higher in the inoculated (T and CT) groups that in the uninoculated (C and CC) groups (*p* < 0.01, Figure 3B). *Funneliformis mosseae* is thought to improve the microenvironment of rhizosphere soil after colonization and symbiosis with soybean roots. SOB richness, abundance, and sulfur metabolism function in rhizosphere soil enhanced, which may boost the accumulation and transport of available sulfur in soil to soybean roots via mycelia and then stimulate the transformation of sulfur in the soil. Compared with the inoculated group, the content of soil available sulfur in the inoculated group was significantly lower, and it fell somewhat compared to the inoculated group and increased at the peak of the infection rate (*p* < 0.05). According to the analysis of the soybean growth process, the content of available sulfur in soil has been steadily decreasing. The soybean plants were in a high-speed growth stage from 45 to 60 days, with a high level of biomass and enzyme synthesis and a high requirement for sulfur. However, during reproduction and maturation in the late growth stage, the need for S reduced, and the sulfur content and distribution in the rhizosphere soil stabilized. According to the findings, AMF mycorrhiza could increase the available sulfur content in rhizosphere soil.

#### 3.2.2. Sulfur content in functional leaves of soybean

The sulfur content in the functional leaves (the upper leaves of soybean plants provide more nutrients than the lower leaves, so we used the leaves of the third branch of the soybean plant counting from the top) of soybean gradually dropped and stabilized during the growth process (Figure 3C). At 30 days, the uncontinuously cropped treatment had significantly higher foliar sulfur content than the continuously cropped treatment (*p* < 0.05). Among the four treatment groups, the sulfur content in the leaves of the T group was the highest, while that of the CC group was the lowest, indicating that continuous cropping caused a deficiency in soil sulfur. Sulfur absorbed by soybean from the soil decreased as well, resulting in a fall in the sulfur content in leaves, which hampered soybean plant growth and development. The sulfur content in the leaves of the inoculated group was significantly higher than that of the uninfected group from 45 to 60 days (*p* < 0.05). It is speculated that *F. mosseae* formed a symbiotic relationship with the soybean roots, and that the exophytic mycelia of the mycelium complex significantly increased the enrichment and absorption of SO_4_^2−^ in the low-solubility and low-density areas of the soybean roots to supplement the sulfur requirement for methionine synthesis and subsequent physiological activities in the rapid growth stage of soybean (*p* < 0.05). Because of the correction of the plants’ overall growth strategy, the physiological stage of functional leaves, S utilization, and sulfur content stabilized between 75 and 90 days. According to the findings, AMF mycorrhiza can increase the available sulfur content in functioning leaves.

#### 3.2.3. Correlation analysis of soil sulfur

According to the correlation analysis of available sulfur in the soil of the four treatments (Figure 3D), following the inoculation of *F. mosseae*, the available sulfur content in the soil of the noncontinuous cropping group increased by 6–9%, whereas it grew by 4–7% in the continuous cropping group. Pearson’s test analysis showed that the correlation coefficient between the two groups was greater than 0.7, which indicated a significant correlation. The sulfur content of soybean functional leaves increased by 4–16% after inoculation with *F. mosseae* and by 4–20% in the continuous cropping group, indicating that the amount and percentage of available sulfur in the soil had a direct effect on the sulfur content of the functional leaves. The inoculation of *F. mosseae* significantly improved the S supply in the soybean rhizosphere soil and plants and indirectly affected the physiological activity of S in related reactions (*p* < 0.05). The results demonstrated that AMF mycorrhiza could increase the content and proportion of available sulfur in rhizosphere soil and improve soybean’s total sulfur nutrition supply.

### 3.3. Analysis of microbial community structure and function in rhizosphere soil

#### 3.3.1. Change in species

**Figure 4:**
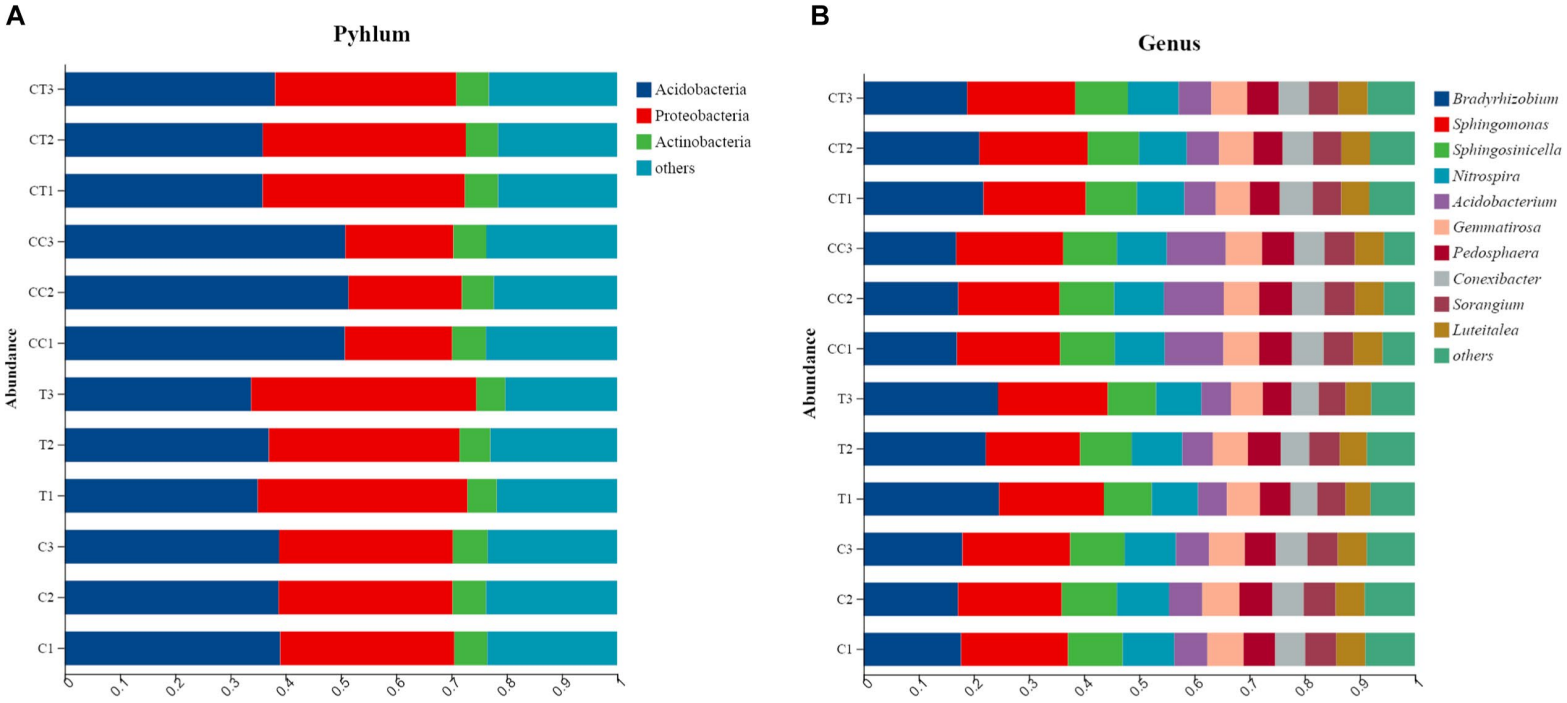
**(A)** Effect of *Funneliformis mosseae* and continuous cropping on the horizontal abundance of the bacterial community in the soil; **(B)** Influence of *F. mosseae* and continuous cropping on the horizontal abundance of the bacterial communities in the experimental soil.

The phylum-level analysis revealed that the microbial flora in the four treatments’ rhizosphere soil consisted of 11 phyla (Figure 4A), and the continuous cropping treatment significantly changed the relative abundance of the two dominant phyla in the soil (*p* < 0.05): the relative abundance of *Acidobacteria* increased and that of *Proteobacteria* decreased. Obviously, the inoculation treatment increased the relative abundance of *Proteobacteria* in the soil. The flora was composed of 12 genera, according to the level analysis (Figure 4B), and continual cropping significantly increased the relative abundance of *Acidobacterium* (*p* < 0.05), while decreasing the relative abundance of *Haliangium*. The inoculation treatment clearly raised *Bradyrhizobium* relative abundance while decreasing the relative abundance of *Sphingomonas*. The results indicate that continuous cropping and inoculation altered the abundance and structure of the soil microbial community.

**Figure 5:**
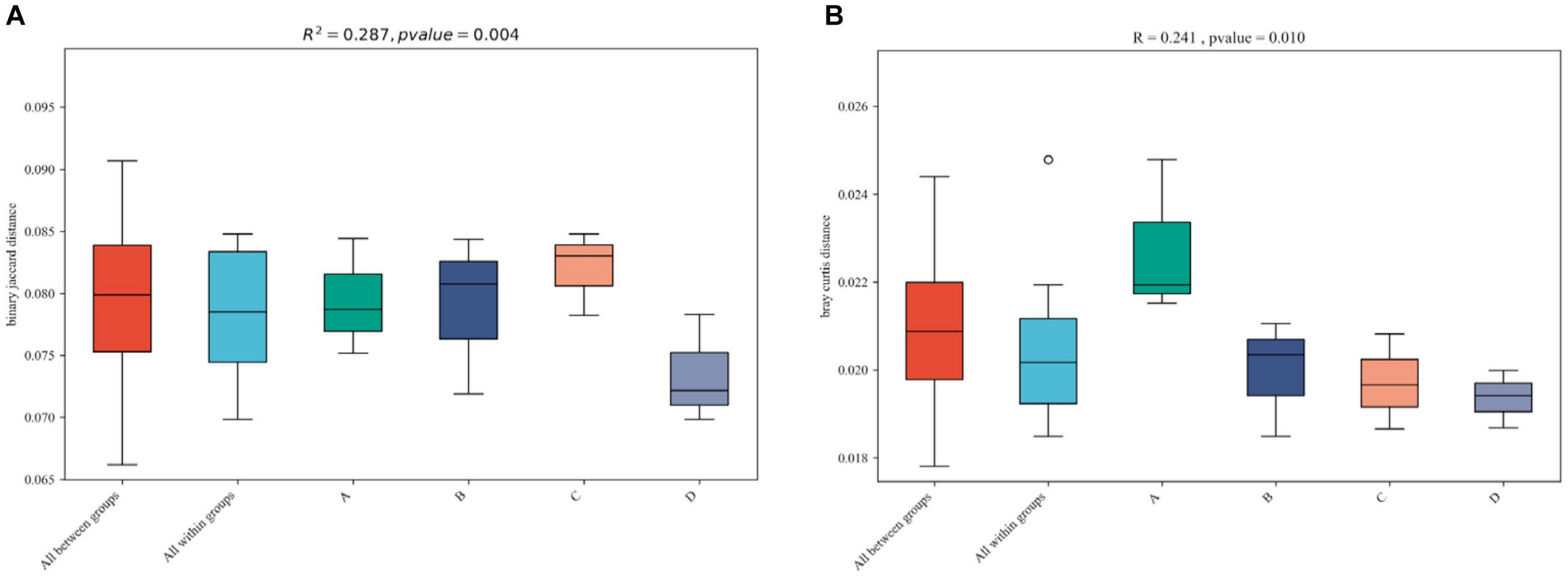
Effects of *Funneliformis mosseae* and continuous cropping on the α diversity of the experimental soil communities, Chao 1 **(A)** and ACE **(B)**.

The Chao1 (Figure 5A) and ACE diversity indices (Figure 5B) were used to assess the α diversity of the four treatment groups. The results of the investigation revealed clear disparities between the continuous cropping and control groups, as well as the inoculation and control groups. The continuous cropping and inoculation treatments significantly changed the diversity of microbial flora in the soil around the roots of continuously cropped soybean (*p* < 0.05).

The nonparametric dissimilarity test (Adonis) was used to evaluate the following factors: noncontinuous and continuous cropping, noninoculation and inoculation with *F. mosseae*. Continuous cropping and inoculation of *F. mosseae* led to noticeable variations in the microbial community structure in the rhizosphere soil of continuously cropped soybean compared to the control group (Table 2).

**Table 2:** Effect of inoculation on the relative abundance of KEGG.

Adonis									
Group		UC		C		NL		L	
		*R*^2^	*p*	*R*^2^	*p*	*R*^2^	*p*	*R*^2^	*p*
Diversity	Phylum	0.133	0.031	0.142	0.028	0.226	0.029	0.242	0.027
	Class	0.142	0.049	0.294	0.044	0.369	0.042	0.285	0.048
	Order	0.182	0.038	0.233	0.039	0.342	0.043	0.504	0.045
	Family	0.076	0.044	0.148	0.033	0.337	0.047	0.395	0.044
	Genus	0.191	0.028	0.242	0.022	0.316	0.029	0.343	0.028

UC, noncontinuous; C, continuous; NL, no inoculation; L, inoculation.

#### 3.3.2. Analysis of functional gene composition and abundance

##### 3.3.2.1. Analysis of functional gene classification

By counting the number of functional genes annotated by KEGG, it was determined that the number of KO sequences in continuously cropped soil was lower than that in noncontinuously cropped soil, the number of KO sequences in inoculated soil was higher than that in noninoculated soil, and the number of KO sequences in inoculated soil was higher than that in continuously cropped soil (Table 3). The number of KEGG pathways in the four treatments was the same, indicating that continuous cropping inhibited microbial functional gene expression in the soybean rhizosphere soil and that inoculation promoted microbial functional gene expression. Continuous cropping altered the structure of the soil microbial flora, reduced population diversity, deteriorated the soil environment, and decreased the number of functional genes. The results suggest that the *F. mosseae* inoculation changed the microbial flora structure and increased the number of functional genes in soil, and that *F. mosseae* was helpful in overcoming the obstacles of continuously cropped soybean.

**Table 3:** Statistics on the number and pathway of functional genes annotated by KEGG.

Sample ID	KO	Average KO	Pathway
C1	6,868	6,834	173
C2	6,854		173
C3	6,813		173
T1	7,055	7046^**^	173
T2	7,002		
T3	7,119		172
CC1	6,537	6536^**^	173
CC2	6,562		173
CC3	6,510		173
CT1	6,969	6,918	173
CT2	6,933		173
CT3	6,924		173

^**^ indicates significant value *p* < 0.05.

**Figure 6:**
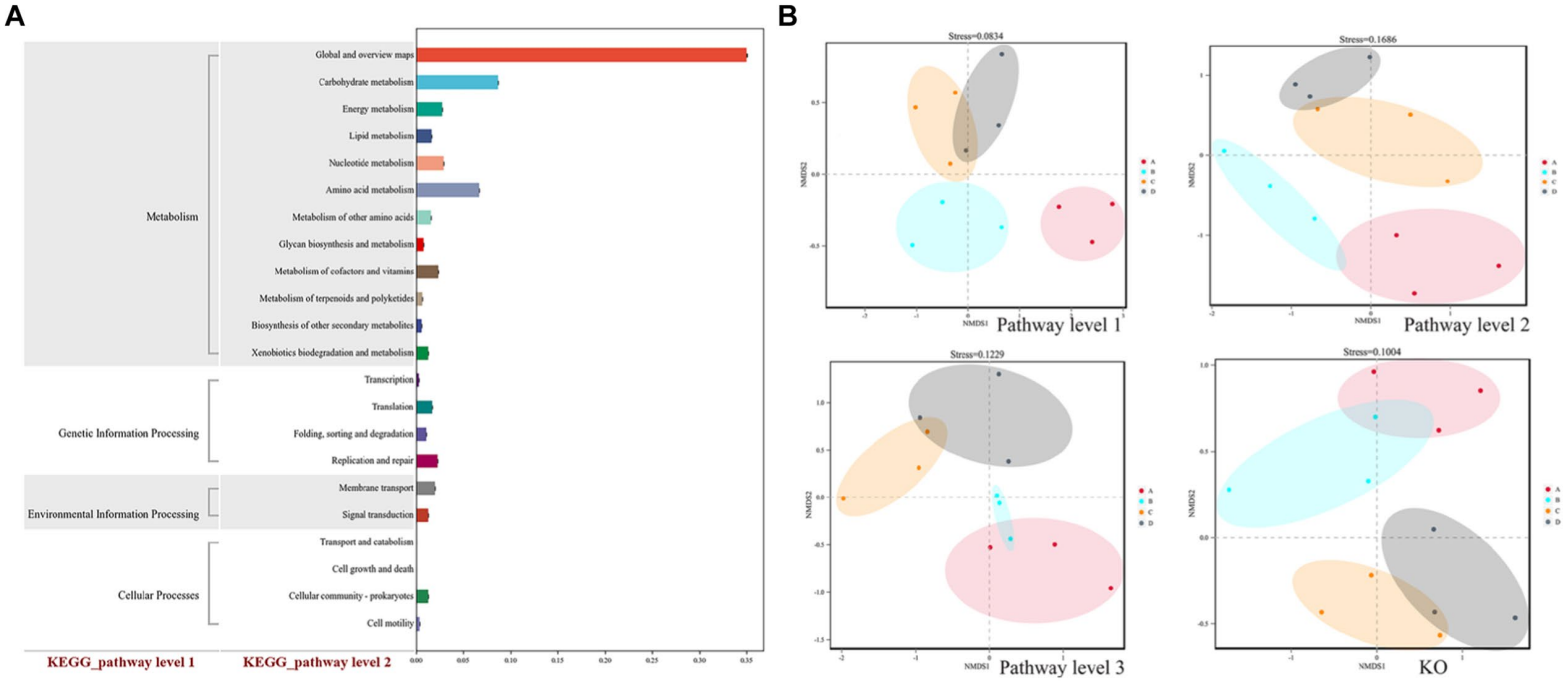
**(A)** Statistics of KEGG function classification and **(B)** NMDS analysis of the relative level of functional genes. Plots A (for control group C), B (representing the noncontinuous inoculation group T), C (for single continuous cropping group CC), and D (for continuous cropping and inoculation group CT). The dots in the NMDS analysis diagram represent one sample, different colors represent different groups, and the distance between two dots indicates the degree of difference. When the stress is less than 0.2, the NMDS analysis has a certain reliability. The closer the distance is on the coordinate diagram, the higher the similarity of the functional gene composition.

The soybean rhizosphere soil microbial community’s functional genes included metabolism, genetic information processing, environmental information processing, and cellular functions (Figure 6A). Further induction and analysis were carried out to see whether the functional gene composition based on KEGG database annotation changed at all levels. The relative content of metabolic functional genes was the highest among the functional genes related to the soil microbial metabolic pathway, with carbohydrate metabolic functional genes having the highest relative content, followed by genes involved in amino acid metabolism, nucleotide metabolism, energy metabolism, cofactors, and vitamin metabolism.

NMDS analysis illustrated four distinct clusters of functional genes in the experimental group, and two clusters clearly separated, indicating that continuous cropping and inoculation treatment significantly changed the composition of functional genes in the control group and treatment group and that inoculation treatment led to the clearly separated overall structure of microbial functional genes in soil compared to the control group (Figure 6B).

**Figure 7:**
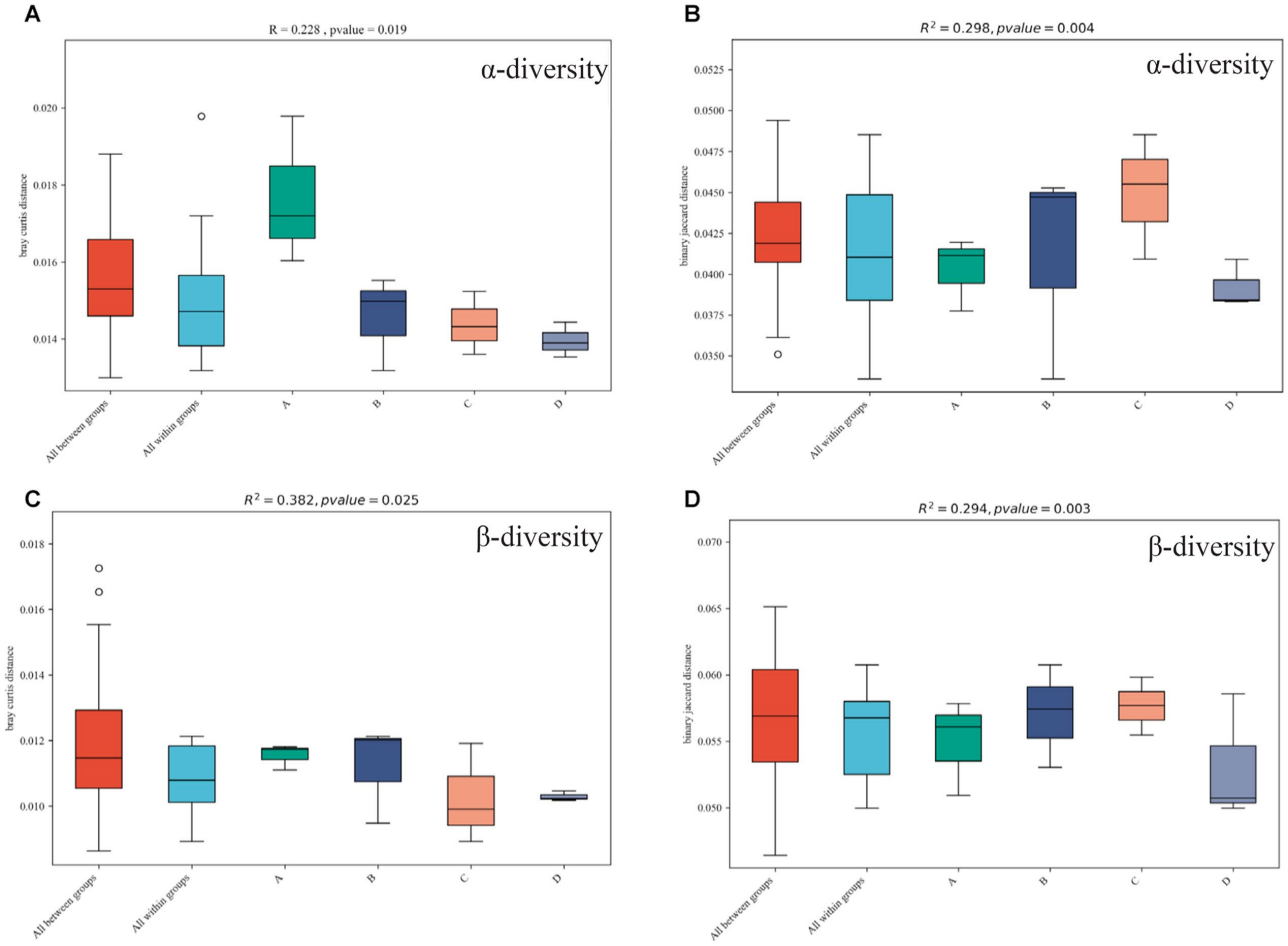
α diversity (**A** is the Chao1 index, **B** is the ACE index) and β diversity (**C** is the Bray–Curtis-Curtis algorithm, **D** is the binary Jaccard algorithm) of functional genes in different treatments annotated by KEGG. Plots A (for control group C), B (representing the noncontinuous inoculation group T), C (for single continuous cropping group CC), and D (for continuous cropping and inoculation group CT).

Chao1 (Figure 7A) and the ACE diversity index (Figure 7B) were chosen to analyze the α diversity of four functional gene structures based on KEGG annotation, and when compared to the control group, continuous cropping and inoculation treatment resulted in obvious differences in the structure of functional genes (*p* < 0.05). The results show that continuous cropping and inoculation significantly changed the α diversity of functional genes in the soybean rhizosphere soil microbial community (*p* < 0.05).

The diversity of the four functional gene architectures based on KEGG annotation was examined using the BrayCurtis (Figure 7C) and binary Jaccard (Figure 7D) algorithms. Continuous cropping and inoculation resulted in significant differences in functional gene composition (*p* < 0.05) as compared to the control group. The result indicates that continuous cropping and inoculation significantly changed the β-diversity of functional genes in the soil microbial community of the soybean rhizosphere (*p* < 0.05).

**Figure 8:**
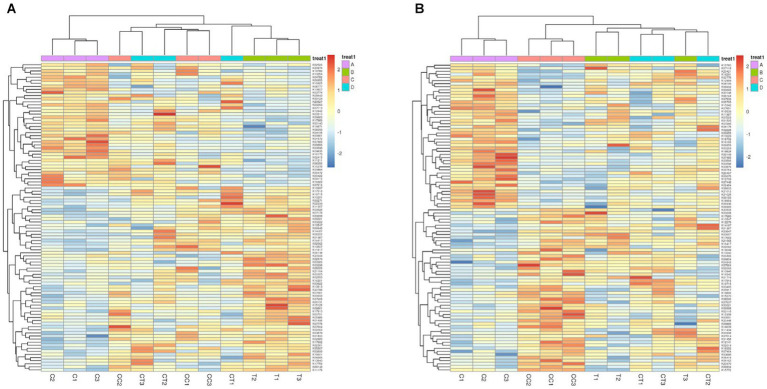
KEGG map of the difference in the abundance of KEGG functional genes, which is expressed by two detection methods (**A** Kruskal–Wallis H test; **B** one-way ANOVA). Plots A: (for control group C), B (representing the noncontinuous inoculation group T), C (for single continuous cropping group CC), and D (for continuous cropping and inoculation group CT).

The relative abundance of functional genes annotated by KEGG was used as the standard to analyze and construct heatmaps using the KruskalWallis H test (Figure 8A) and one-way ANOVA (Figure 8B). The functional genes of the four treatment groups displayed prominent expression tendencies. Compared to the control group, the expression activity of stress resistance genes (e.g., disease resistance) was increased in the continuous cropping treatment, and minerals such as N, P, and S were discovered in the inoculated treatment group. The findings showed that AMF could improve the expression activity of genes involved in mineral element absorption in soybean roots.

##### 3.3.2.2. Difference analysis of functional genes

**Figure 9:**
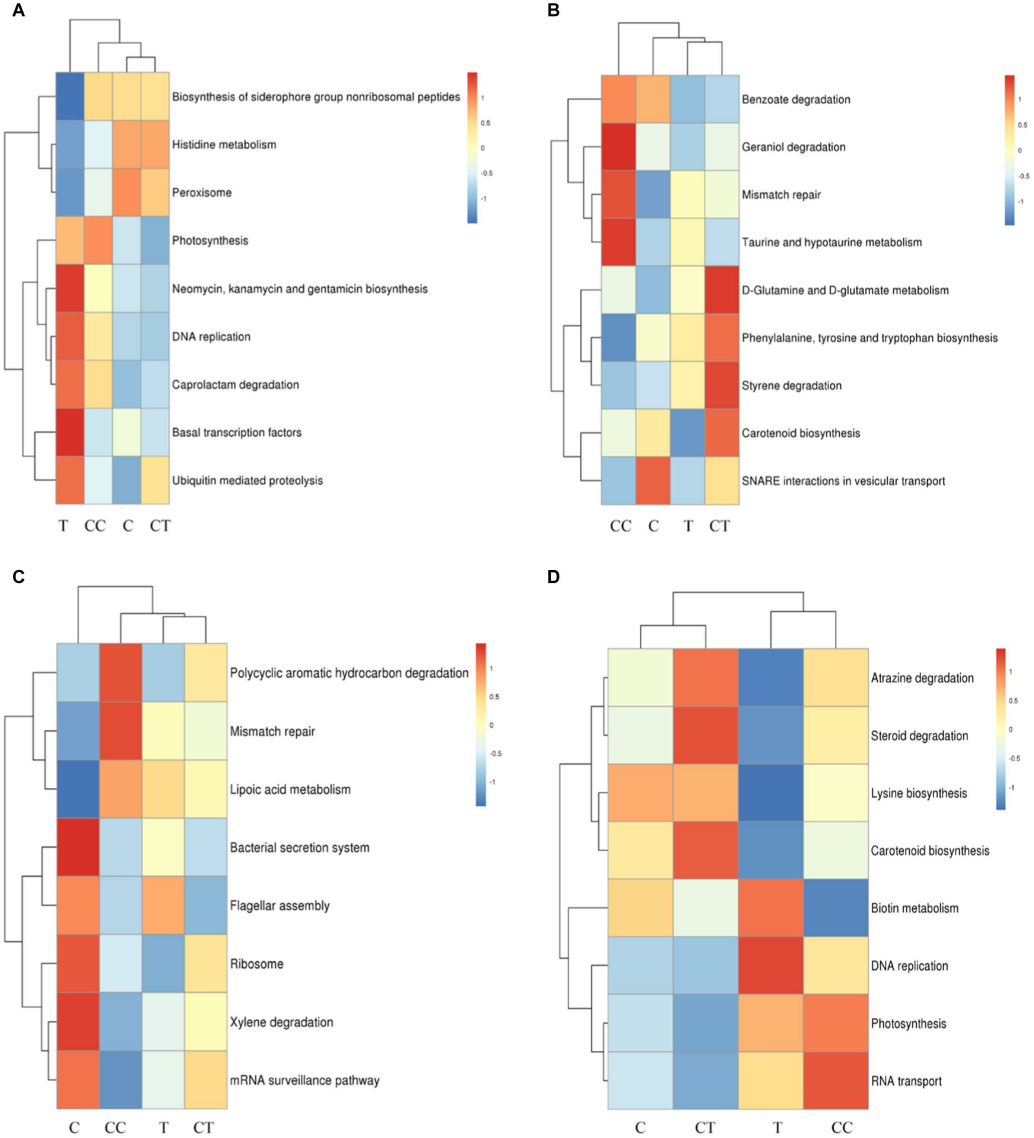
Difference analysis of functional genes. **(A)** Comparison of the differential functional genes of group C and T; **(B)** Comparison of the differential functional genes of group CC and CT; **(C)** Comparison of the differential functional genes of group C and CC; **(D)** Comparison of the differential functional genes of group T and CT.

The functional genes of the iron carrier group, as well as those involved in nonribosomal peptide biosynthesis, histidine metabolism, and peroxisome, were substantially higher in the C treatment than in the T treatment (*p* < 0.05), as shown in Figure 9A. The functional genes involved in photosynthesis, neomycin, biosynthesis of kanamycin and gentamicin, DNA replication, caprolactam degradation, basic transcription factors, and ubiquitin-mediated proteolysis were significantly higher in the T treatment than in the C treatment (*p* < 0.05). *Funneliformis mosseae,* as an artificial AMF, is thought to have been backward in the invading soybean rhizosphere soil group, promoting the expression of the resistance-related functional genes in the host plant soybean and rhizosphere microorganisms.

As shown in Figure 9B, the functional genes involved in the bacterial secretion system, flagella assembly, ribosome, mRNA monitoring pathway, and xylene degradation were significantly higher in the C treatment group than in the CC treatment group (*p* < 0.05), and the functional genes involved in polycyclic aromatic hydrocarbon degradation, mismatch repair, and lipoic acid metabolism were significantly higher in the CC treatment group than in the C treatment group (*p* < 0.05). Continuous cropping is thought to harm the ecological environment of the soil surrounding soybean roots and diminish the overall metabolic level of most indigenous microorganisms.

Compared to the CT treatment (Figure 9C), the functional genes involved in benzoic acid degradation, geraniol degradation, mismatch repair, taurine, and hypotaurine metabolism were significantly higher in the CC treatment group (*p* < 0.05), while the functional genes involved in d-glutamine and d-glutamic acid metabolism, phenylalanine, tyrosine and tryptophan biosynthesis, styrene metabolism and carotenoid biosynthesis were significantly higher in the CT treatment group than in the CC treatment group (*p* < 0.05). Continuous cropping is thought to have reduced soil microbial activity, resulting in a decline in microbial synthesis function, while the genes responsible for microbial metabolism function obviously increased following inoculation. The result indicated that inoculation with *F. mosseae* improved the microbial community function of soybean rhizosphere soil and alleviated the continuous cropping problem.

Function genes such as those involved in RNA transport, photosynthesis, DNA replication, and biotin metabolism were significantly higher in the T treatment group than in the CT treatment (*p* < 0.05, Figure 9D), and atrazine degradation, steroid degradation, lysine biosynthesis and carotenoid biosynthesis in the CT treatment group were significantly higher than those in the T treatment group (*p* < 0.05). The functional genes that can boost the synthesis of growth chemicals and breakdown hazardous substances are thought to have changed considerably (*p* < 0.05) after the continuously cropped soil was inoculated with *F. mosseae*.

The following factors were tested using a nonparametric dissimilarity test (Adonis): noncontinuous cropping, continuous cropping, non-inoculation, and inoculation. Continuous cropping and *F. mosseae* inoculation led to significant differences in the composition, structure, and quantity of functional genes in the soil microbial community between the control group and the experimental group (*p* < 0.05, Table 4).

**Table 4:** Statistical analysis of the relative abundance of functional genes annotated by KEGG.

Adonis									
Group		UC		C		NL		L	
		*R*^2^	*p*	*R*^2^	*p*	*R*^2^	*p*	*R*^2^	*p*
KEGG	Level 1	0.572	0.034	0.239	0.032	0.399	0.031	0.257	0.029
	Level 2	0.593	0.027	0.371	0.041	0.348	0.023	0.549	0.038
	Level 3	0.522	0.048	0.433	0.029	0.942	0.045	0.552	0.042
	KO	0.491	0.036	0.542	0.031	0.243	0.039	0.543	0.027

UC, noncontinuous; C, continuous; NL, no inoculation; and L, inoculation.

#### 3.3.3. Analysis of differences between genus-level groups and differences in functional genes in SOB

##### 3.3.3.1. Analysis of differences among horizontal groups of the SOB genus

**Figure 10:**
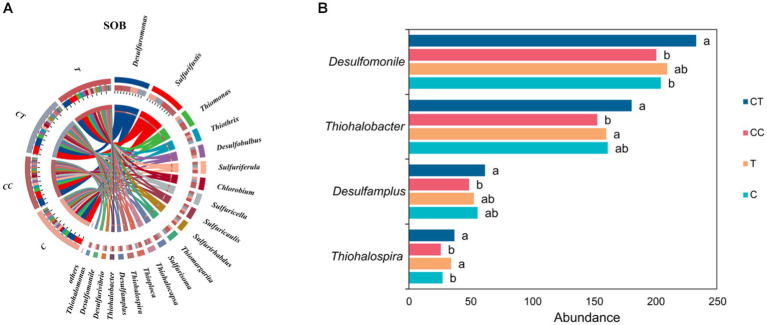
**(A)** SOB belongs to the horizontal difference (the arc length represents abundance); **(B)** Difference in SOB abundance of dominant species.

According to the species abundance analysis of SOB in the four treatment groups (Figure 10A), *Desulfuromonas* had the highest abundance, followed by *Thiobacillus*, *Thioalkalivibrio*, *Beggiatoa,* and *Thiomonas*. Most SOB genera, such as *Desulfuromonas, Thiobacillus, Beggiatoa, Thiomonas, Thiothrix*, *Chlorothiobacillus,* and *Desulfobulbus*, has larger abundances in the CT treatment group than in the C and T groups. *Thiohalobacter, Thiohalospira, Desulfamplus,* and *Desulfomonile* were considerably more abundant in the CT group than in the CC group (*p* < 0.05). The horizontal abundance of SOB was lowest in the CC group among the four treatments, and the abundance of *Thiohalospira* in the T group was significantly higher than that in the C group (*p* < 0.05, Figure 10B). It is hypothesized that the inoculation of *F. mosseae* improved the soil microecological environment, microbial diversity, soil enzyme activity, and metabolic function of sulfide bacteria, resulting in an increase in the abundance of soil SOB flora.

##### 3.3.3.2. Differential analysis of functional genes of SOB genus horizontal sulfur

Soil SOB exhibits specificity and continuity in the expression of the sulfur function. Following the exclusion of plant genes, a difference analysis of the sulfur functional genes of SOB in the four treatment groups showed that, when compared to the control group, the expression level of the sulfur functional genes in the inoculated group was higher, but that in the continuous cropping group was apparently lower (*p* < 0.01).

**Figure 11:**
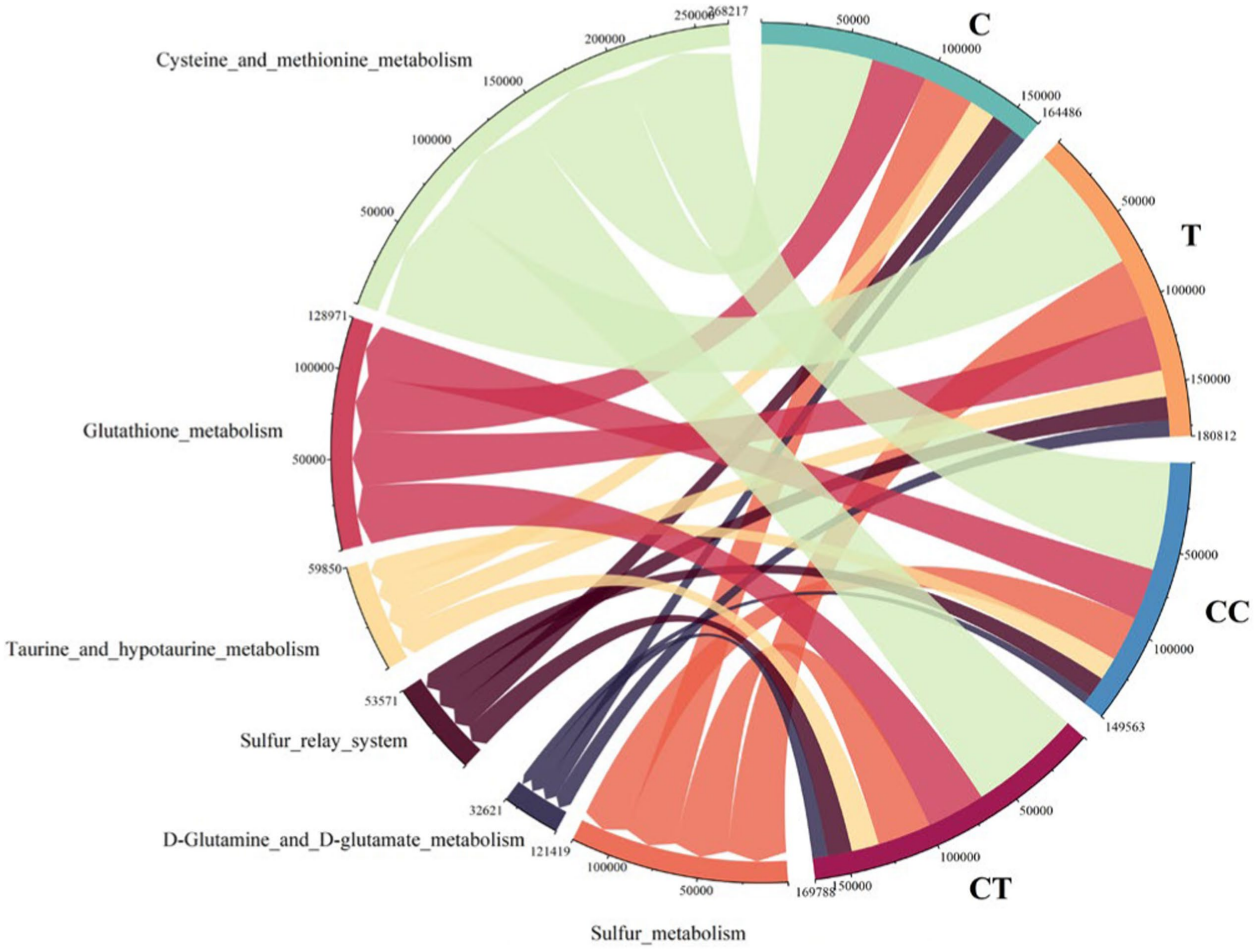
SOB difference in SOB functional genes (the arc length represents abundance).

Cysteine and methionine metabolism had the highest expression abundance among the SOB functional genes (Figure 11), followed by Glutathione metabolism, Sulfur metabolism, taurine and hypotaurine metabolism, Sulfur relay system, and d-glutamine and d-glutamate metabolism. The sulfur-related reaction in the SOB population is thought to be primarily the absorption and utilization of accessible sulfur in the natural environment. Functional gene expression levels in CT and C were similar but significantly different from those in CC (*p* < 0.05). It is speculated that AMF can restore the expression of restricted SOB functional genes.

### 3.4. Soybean biomass

**Figure 12:**
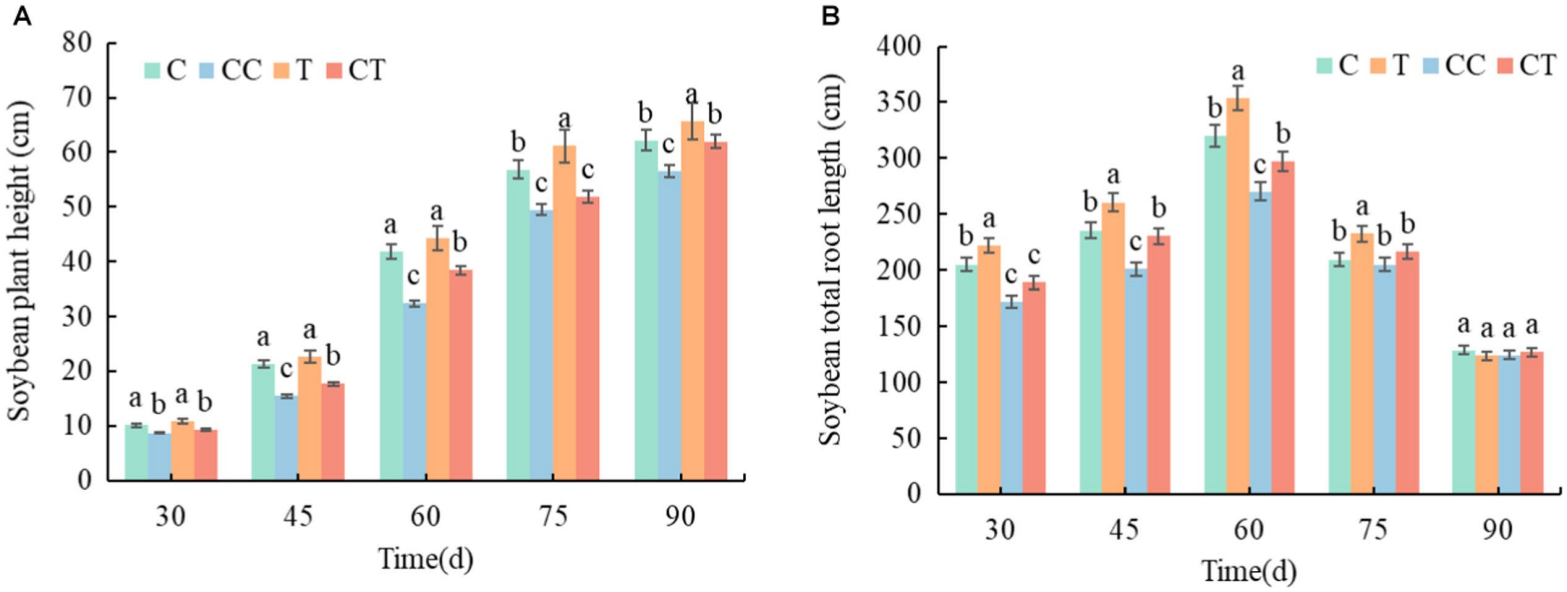
Changes in plant height and root length and growth of soybean plants between treatments. **(A)** Time variation trend of soybean plant height in four samples and **(B)** time variation trend of total root length of soybean in four samples.

During the growth period of soybean, the plant height of the four treatments increased (Figure 12A), whereas total root length increased from emergence to and then gradually decreased after 60 days (Figure 12B). In comparison to the other groups, the inoculated group outperformed the uninfected group, while the continuous cropping group was worse than the noncontinuous cropping group. The T group had obvious advantages and was significantly different from the other three groups during the growth period, although the CT group had no significant difference from the C group after 60 days (*p* < 0.05).

**Figure 13:**
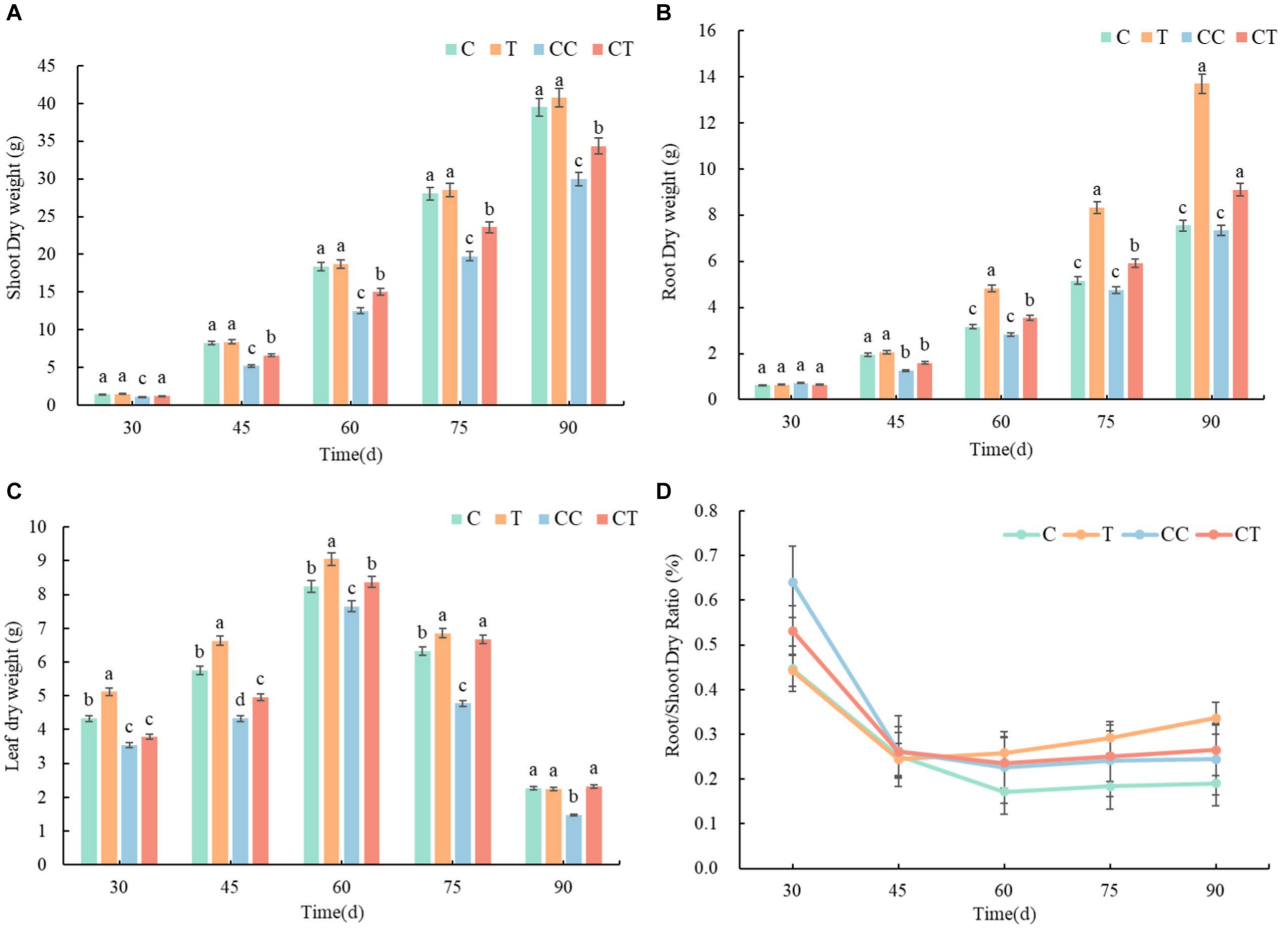
Changes in dry weight and growth of soybean plants between treatments. **(A)** Dry weight of aboveground parts of four samples, **(B)** dry weight of roots of four samples, **(C)** dry weight of leaves of four samples, and **(D)** root-shoot ratio of four samples.

During the growing period of soybean, the biomass of plants in all four treatments, and the biomass accumulation obviously accelerated after 30 days and remained steady after 90 days. The root-shoot ratio of plants decreased in all treatments, and the root-shoot ratio remained unchanged after 60 days (Figure 13). According to the examination of the AMF infection rate of soybean roots, the root-shoot ratio of the inoculated group was lower than that of the uninfected group from emergence to 45 days. It was proposed that *F. mosseae*’s nutrition mode was parasitic and would absorb the nutrients from the attached root tissue in the early self-development stage, causing stress and limiting the development of the soybean roots. The root-shoot ratio of the inoculated group was considerably higher than that of the uninfected group from 45 to 90 days (*p* < 0.05). After 30 days, the overall infection rate of the injected group was substantially greater than that of the uninfected group (*p* < 0.05), when combined with the study of the AMF infection rate during the growth phase. When the root infection rate of the inoculated group exceeded 80% after 60 days, the inoculated group had marked advantages in plant growth and root development compared with the uninfected group, indicating that *F. mosseae* significantly promoted the growth and nutrient absorption of soybean plants. The dry weights of the crowns, roots, and plants in the noncontinuous cropping group were substantially higher than those in the continuous cropping group over the growth period (*p* < 0.05), according to an examination of the continuous cropping parameters of soybean. The main reasons were an increase in the abundance of pathogenic bacteria in the soil microbial community and a decrease in the contents of toxic substances and mineral elements secreted by the soybean roots, resulting in the destruction of the soil ecosystem and environmental stress during soybean growth. According to the comprehensive analysis of the AMF infection rate and the continuous cropping factors of the soybean roots, the dry weight indices of the inoculated group were significantly better than those of the uninoculated group during the entire growth period, and the whole continuous cropping group was lower than that of the noncontinuous cropping group (*p* < 0.05), which indicated that inoculation with *F. mosseae* effectively improved the root growth obstacle caused by continuous cropping. After the roots of the soybean plants formed mycorrhizae, their own growth and nutrient absorption greatly improved, as did their stress resistance and adaptability to the stress environment improved significantly (*p* < 0.05).

## 4. Discussion

### 4.1. Continuous cropping reduced population diversity

Continuous soybean cropping can cause problems such as the accumulation of toxic and harmful substances in root exudates, soil hardening, soil nutrients imbalance, and so on, resulting in changes in the soil microecological environment, a restructuring of the soil microbial community structure, a reduction in population diversity, a reduction in soil enzyme activity, and a reduction in the number of functional genes. Soybean is a crop that thrives on S, and continuous cropping consumes large amount of soil S, but the soil S is deplete and its S metabolic function is weakened, causing the soil sulfur cycle to be hampered (Liu et al., 2021). Inoculation with *F. mosseae* can improve the soil microecological environment, increase microbial diversity, alleviate the toxic effect of root exudates, improve soil enzyme activity, promote the transfer of nutrients enhance biosynthesis, assist soil microorganisms in recovering their activities, and increase the number of functional genes in soil (Fernandes et al., 2021).

### 4.2. Intergroup difference analysis at the SOB genus level

The soil hardens and gets acidified as a result of the long-term closure of soybean constantly cropped soil and the use of compound fertilizers such as potassium phosphate/potassium nitrate (Deng et al., 2021; Fernandes et al., 2021). Under acidic conditions, the available S in the soil is easily converted to elemental sulfur or hydrogen sulfide, which leads to sulfur loss from the soil and a downward shift in sulfur distribution, which is not conducive to the survival and development of soil SOB groups in the cultivated area around the roots (Qi et al., 2021; Tang et al., 2022).

We discovered that the pH of the soil was lower in the continuous crop treatment group, and the sulfur distribution was closer to the deep layer, which benefited the proliferation of sulfur-oxidizing bacteria. Therefore, the continuous cropping group had a plethora of desulfurization and sulfur-oxidizing fungi. In this study, we found that soil aeration was improved by inoculation with AMF. The sulfur attraction enrichment and assimilation capacity of the mycorrhizal symbiosis was greater than that of a single soybean root system. Inoculation with *F. mosseae* also improved the nutrient supply as well as the physical and chemical properties of the soil around the roots, which aided the growth of sulfide fungi in the middle and higher layers. *F. mosseae* will expand the coverage of the root zone, resulting in additional genera participating in photosynthetic sulfide oxidation in the microsurface layer and interaction with the rhizosphere flora (Wu et al., 2020; Deng et al., 2021; Guo et al., 2021; Sun et al., 2021; Wang et al., 2021). SOB abundance was higher in the CT group, and PSB abundance was higher in the T and CT groups was higher than in the C and CC groups.

### 4.3. Intergroup difference analysis of functional genes

#### 4.3.1. Intergroup difference analysis of full functional genes

Continuous cropping will cause the entire soil structure to harden and solidify, greatly reducing the efficiency of water and gas circulation, and increasing the environmental pressure for the survival of the rhizosphere microbial population, affecting its growth, migration, reproduction, and other activities (Wang et al., 2021a). Compared to the noncontinuous cropping group, the expression level of the overall functional genes decreased. In addition, the relative abundance of the major functional genes, which mainly included the following two categories listed below, declined: disease-resistance-related functional genes (isocitrate lyase, fibronectin, apolipoprotein D and lipoprotein family proteins, guanylate cyclase, acetyl-CoA synthetase, and alanine dehydrogenase etc.), and nutrition-related functional genes (filamentous haemagglutinin, metabolite transporter, and phosphoribosyl).

When continuously cropped soil was inoculated with *F. mosseae*, the relative abundance of soil functional genes, including the TPR domain protein, ABC type no characteristic transport system, trypsin, dithiooxamide biosynthetic protein, and I type phosphodiesterase/nucleotide pyrophosphatase, DNA polymerase, dopa 4,5-dioxygenase, polysaccharide biosynthetic protein, basic cell division protein and transcription regulator, increased or returned to the level of the control group (noncontinuous cropping), and the whole group.

#### 4.3.2. Intergroup difference analysis of sulfur functional genes

To establish the sulfur cycle, plant and animal residues and excretions in the soil must be degraded into sulfur components by soil microorganisms and released back into the soil or the environment. Continuous cropping reduced the abundance of small microbial flora and weakened functional genes related to sulfur metabolism, indicating that continuous cropping reduced the activity of sulfur bacteria in soil and hampered the sulfur cycle (Dias-Arieira et al., 2021; Guo et al., 2021).

In the present study, we found that inoculation of AMF in continuous cropping soil increased the total and relative abundance of genes related to the sulfur transport system, sulfur metabolism, cysteine and methionine metabolism, glutathione metabolism, thiamine metabolism, and lipoic acid metabolism, indicating that the mycorrhizal structure improved the mineral circulation pathways in the intermycelial region after *F. mosseae* intervened in the soil microcosm (Wang et al., 2021b) and increased the utilization of sulfur substrates and sulfur metabolites by SOB. Simultaneously, *F. mosseae mycelia* produced a signal that promoted the miragtion of sulfide bacteria in the soil to the hyphal region. As a result inoculation with *F. mosseae* stimulated the soil sulfur cycle, oxidized more fixed sulfide into inorganic sulfur, improved the soil sulfur conditions, and increased the availability of sulfur to the soybean plants.

### 4.4. SOB and RDA of soil environmental factors

**Figure 14:**
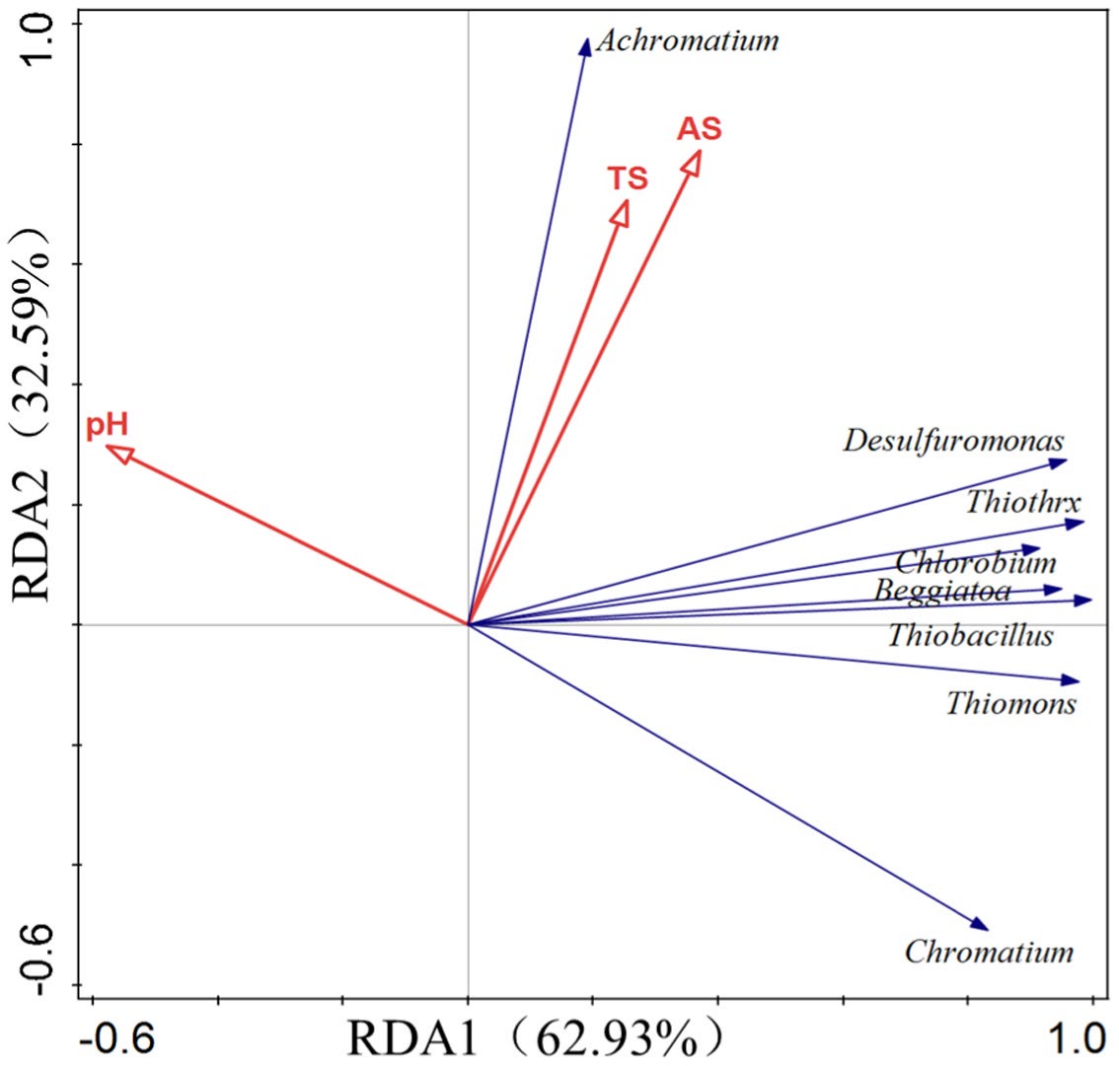
Redundancy analysis (RDA) showed the relationship between the abundance of microbial species participating in S cycle and S in the experimental soil. The direction and size of the arrow indicate the relationship between gene expression intensity and soil factors. TS, soil total sulfur; and AS, soil available sulfur.

The RDA approach was utilized to analyze SOB in soil as well as soil environmental parameters such as total sulfur (TS), available sulfur (AS), and pH value. The two-axis sorting results showed that all calculation models passed the significance test (*p* < 0.01). The pH value was adversely connected with the majority of the SOB bacteria, such as *Desulfuromonas*, *Thiobacillus, Beggiatoa, Thiomonas, Thiothrix, Chlorobium, Desulfobulbus*, and *Chromatium* (purple sulfur bacteria). It is believed that SOB flora will influence the soil sulfur content in the following two ways: the acidification of soil to accelerate the conversion efficiency of available sulfur or the oxidization or reduction of sulfide to generate a new form of available sulfur (Figure 14).

## 5. Conclusion

*Funneliformis mosseae* inoculation increased the available sulfur content in the rhizosphere soil of continuously cropped soybean, improved sulfur absorption by soybean, promoted the growth of soybean plants, and alleviated the obstacles of continuously cropped soybean. Inoculation with *F. mosseae* increased sulfur-related metabolic functions, such as sulfur metabolism, thiamine metabolism, and lipoic acid metabolism. Most SOB genera, such as *Desulfuromonas, Thiobacillus, Beggiatoa, Thiomonas, Thiothrix, Chlorobium*, and *Desulfobulbus*, increased in abundance in the community, while *Thiohalobacter, Thiohalospira, Desulfamplus*, and *Desulfomonile* grew significantly (*p* < 0.05). In the soil community, the overall abundance and relative abundance of functional genes associated with the sulfur transport system, sulfur metabolism, cysteine and methionine metabolism, glutathione metabolism, thiamine metabolism, and lipoic acid metabolism increased.

## Data availability statement

The original contributions presented in the study are included in the article/supplementary material, further inquiries can be directed to the corresponding author.

## Author contributions

YM and BC designed the study. YM, DC, XC, and YW carried out the experiments. YM performed data mining. YM and DC wrote the publication. All authors contributed to the article and approved the submitted version.

## Funding

This work was supported by grants from the National Natural Science Foundation of China (No. 31972502).

## Conflict of interest

The authors declare that the research was conducted in the absence of any commercial or financial relationships that could be construed as a potential conflict of interest.

## Publisher’s note

All claims expressed in this article are solely those of the authors and do not necessarily represent those of their affiliated organizations, or those of the publisher, the editors and the reviewers. Any product that may be evaluated in this article, or claim that may be made by its manufacturer, is not guaranteed or endorsed by the publisher.
